# LASSO-derived nomogram prediction model for lymph node metastasis in colorectal cancer: a retrospective analysis

**DOI:** 10.7717/peerj.19148

**Published:** 2025-04-14

**Authors:** Xiyun Quan, Yi Deng, Zhimin Liu, Zhenqin Gao, Huimei Yi, Ming Li

**Affiliations:** 1 Department of Pathology, Zhuzhou Hospital Affiliated to Xiangya School of Medicine, Central South University, Zhuzhou, Hunan, China; 2 Neurosurgery, Zhuzhou Hospital Affiliated to Xiangya School of Medicine, Central South University, Zhuzhou, Hunan, China; 3 Health Management Center, Zhuzhou Hospital Affiliated to Xiangya School of Medicine, Central South University, Zhuzhou, Hunan, China

**Keywords:** Colorectal cancer, Lymph node metastasis, Clinical nomogram, Prediction model, LASSO logistic algorithm

## Abstract

**Objective:**

This study aims to develop a prediction model for lymph node metastasis (LNM) in colorectal cancer (CRC) patients using common clinicopathologic data and a nomogram. The model seeks to uncover correlations between LNM and clinical indicators, providing an effective tool to identify high-risk patients, aiding clinical decision-making, and enhancing patient prognosis.

**Methods:**

We conducted a retrospective analysis of CRC patients diagnosed between January 2021 and December 2023 at Zhuzhou Hospital Affiliated to Xiangya School of Medicine, Central South University. Risk predictors for LNM were identified through comparative analysis and Least Absolute Shrinkage and Selection Operator (LASSO) logistic regression. Nomograms were then utilized to predict the probability of metastasis, and their performance was assessed using calibration curves, receiver operating characteristic (ROC) curves, and decision curve analysis.

**Results:**

The study comprised 869 CRC patients, with 435 cases allocated to the training set and 434 cases to the validation set. First, 12 potential risk factors for LNM in CRC patients were identified through comparative analysis in the training set. Next, nine independent predictors (T stage, vascular tumor thrombus, PMS2, MSH2, KRAS, BRAF, PIK3CA, leukocyte, and neutrophil) of LNM occurrence were refined using LASSO regression and multivariate logistic regression models. Subsequently, a clinical nomogram was developed based on these independent predictors of LNM. The nomogram exhibited a C-index of 0.751 (95% CI [0.728–0.774]), indicating its robust predictive value, which was further validated in the independent validation set.

**Conclusion:**

T stage, vascular tumor thrombus, PMS2, MSH2, KRAS, BRAF, and neutrophil emerged as significant risk factors for LNM in CRC, while leukocytes appeared to be protective. These findings emphasize the importance of comprehensive risk assessment and personalized therapeutic strategies in CRC management.

## Introduction

Colorectal cancer (CRC) is the third most common primary malignancy and the second leading cause of cancer deaths worldwide. In 2020, there will be about 1.9 million new cancer cases and 935,000 deaths (Sung et al., 2021). Advances in multimodal therapies and new chemotherapeutic drug treatments have reduced CRC mortality at a rate of 2% per year over the past decade, but CRC remains a deadly disease (Spaander et al., 2023). In addition to an aging population, obesity, unhealthy eating habits, alcohol consumption, and smoking increase the risk of CRC (Cervantes et al., 2023; Malvezzi et al., 2018). Approximately 20% of patients with a first clinical diagnosis of colon cancer are associated with metastases (Rumpold et al., 2020).

Lymph node metastasis (LNM) represents the most prevalent metastatic pathway for CRC. The prognosis for patients with CRC associated with LNM is usually poor: the 5-year survival rate is 31% (Hu et al., 2021). After resection by radical surgery or adjuvant therapy, 30% of patients with metastatic CRC will still recur, with approximately 20% of lymph node-negative patients recurring 5 years after initial surgery (Brask-Thomsen & Love, 2022; Carrara et al., 2020; Duineveld et al., 2016).

According to clinical treatment guidelines based on histopathologic examination, CRC patients with low-risk lymph node metastases can be locally resected by endoscopic submucosal dissection (ESD), while high-risk patients require more extensive radical resection (Ikematsu et al., 2013). However, these guidelines can only accurately predict lymph node status in 8–16% of patients, with the vast majority (>70%) of LNM-negative patients undergoing unnecessary additional surgery (Siegel et al., 2023; Tanaka et al., 1995; Zhuang et al., 2023). Therefore, timely identification of patients at high risk of lymph node metastasis through the establishment of an effective prognostic model is essential for prolonging the survival and significantly improving the overall prognosis of CRC patients.

With the advancements in machine learning and statistical techniques, constructing predictive models using clinical data has emerged as a potent tool aiding physicians in swiftly and accurately assessing patient risk. In this study, we will employ the Least Absolute Shrinkage and Selection Operator (LASSO) logistic algorithm to model clinical data from CRC patients, aiming to generate a clinical nomogram to predict the risk of LNM in CRC. The LASSO logistic algorithm automatically selects and sparsifies features, aiding in the identification of clinical features significantly impacting LNM. Consequently, this approach enhances the predictive performance and interpretability of the model.

In this study, we conducted a large-sample retrospective analysis to gather common clinicopathological data. Subsequently, we developed a nomogram prediction model for LNM in CRC patients. The objective was to elucidate the relationship between LNM and commonly monitored clinical indicators in CRC patients. We aimed to devise an effective nomogram capable of identifying patients at high risk of LNM, thereby facilitating clinical decision-making and enhancing patient prognosis.

## Methods

### Patients

Data spanning from January 2021 to December 2023 were collected by researchers at Zhuzhou Hospital Affiliated to Xiangya School of Medicine, Central South University. Inclusion criteria encompassed patients with a first diagnosis of CRC during this period. Demographic variables such as age and gender were required, alongside hematological testing indicators including white blood cells, red blood cells, platelets, hemoglobin, neutrophils, lymphocytes, red blood cell distribution width, mismatch repair (MMR), and CRC tumor markers (MLH1, PMS2, MSH2, MSH6, KRAS, BRAF, NRAS, PIK3CA). Additionally, patients underwent colorectal tumor resection either during their first hospitalization or at a later stage of resection in cases with distant metastases. Detailed pathological data including size, gross staging, histologic type, tumour, node, metastasis (TNM) stage, vascular tumor thrombus, and nerve involvement were obtained. The confirmed diagnosis of CRC required validation through at least two imaging examinations or histopathologic diagnosis. Exclusion criteria comprised incomplete information, lack of essential clinicopathological factors, receipt of adjuvant treatments before obtaining pathological information, and presence of other malignant tumors. Ultimately, 869 patients were included for studying diagnostic risk factors in CRC patients with LNM. In the context of CRC, N0 Indicates no regional LNM, while stages N1 and N2 signify LNM. Randomization divided CRC patients into a training set (435 patients) and a validation set (434 patients) using a random number generation algorithm. Specifically, we used the set.seed(123) function in R to ensure reproducibility, and the sample() function was applied to randomly allocate 50% of the patients to the training set and the remaining 50% to the validation set. The randomization was performed without replacement, ensuring that no patient appeared in both sets. The training set was utilized for constructing the nomogram, while the test set was used for validation purposes. This study was approved by the Ethics Committee of Zhuzhou Hospital Affiliated to Xiangya School of Medicine, Central South University, and the ethical approval number was KY2024027-01. We received a waiver of the need for informed consent from participants of our study.

### Statistical analysis

Frequency data were presented as counts (percentages), and differences between groups were assessed using the chi-square test. The normality of continuous data was evaluated using the Kolmogorov–Smirnov method. Data that met the criteria of normal distribution and homogeneity of variances were described as Mean ± SD, and independent samples *t*-test was used for comparisons between groups. Non-normally distributed data or those with uneven variances were described using the median with an interquartile range (M(Q1, Q3)). LASSO and binary logistic regression models were utilized to construct the clinical nomogram. All tests were two-sided with a significance level of *α* = 0.05, where differences were considered statistically significant when *P* < 0.05. Statistical analyses were performed using SPSS 26.0 and R (v 4.2.1; R Core Team, 2022) software.

## Results

### Comparison of clinical factors between training and validation sets

Following the predefined inclusion and exclusion criteria, a total of 869 patients were enrolled in the study, with 435 assigned to the training set and 434 to the validation set using a randomized method. Examination of Table 1 indicated no significant disparities in clinical characteristics between the two sets, suggesting the robustness of the randomization process. The number of all CRC patients with LNM was 380 (43.73%), including 196 (45.06%) in the training set and 184 (42.40%) in the validation set.

**Table 1:** Comparative analysis of clinical and pathological characteristics among CRC patients in the training and validation sets.

**Clinical variables**	**Training set (*n* = 435)**	**Validation set (*n* = 434)**	**Total (*n* = 869)**	**Statistics**	***P***
**Age (years)**	61 (52,69)	63 (55,70)	62 (54,70)	−1.262	0.207
**Gender**				0.056	0.814
Male	265 (60.92)	261 (60.14)	526 (60.53)		
Female	170 (39.08)	173 (39.86)	343 (39.47)		
**Tumor size (cm)**	4.5 (3.5,5.5)	4.5 (3.5,6)	4.5 (3.5,5.9)	−0.961	0.336
**Tumor site**				1.452	0.693
Rectum	194 (44.60)	183 (42.17)	377 (43.38)		
Left colon	126 (28.97)	133 (30.65)	259 (29.80)		
Right colon	92 (21.15)	100 (23.04)	192 (22.09)		
Other	23 (5.29)	18 (4.15)	41 (4.72)		
**General classification**				3.684	0.159
Ulcer type	295 (67.82)	281 (64.75)	576 (66.28)		
Raised type	140 (32.18)	150 (34.56)	290 (33.37)		
Other	0 (0)	3 (0.69)	3 (0.35)		
**Histological type**				3.328	0.650
Poorly differentiated adenocarcinoma	12 (2.76)	13 (3.00)	25 (2.88)		
Moderately-poorly differentiated adenocarcinoma	73 (16.78)	57 (13.13)	130 (14.96)		
Moderately differentiated adenocarcinoma	321 (73.79)	330 (76.04)	651 (74.91)		
High-moderate differentiated adenocarcinoma	9 (2.07)	10 (2.30)	19 (2.19)		
High differentiated adenocarcinoma	5 (1.15)	9 (2.07)	14 (1.61)		
Other	15 (3.45)	15 (3.46)	30 (3.45)		
**T stage**				3.583	0.310
T1	18 (4.14)	18 (4.15)	36 (4.14)		
T2	62 (14.25)	74 (17.05)	136 (15.65)		
T3	296 (68.05)	299 (68.89)	595 (68.47)		
T4	59 (13.56)	43 (9.91)	102 (11.74)		
N stage				2.318	0.314
N0	239 (54.94)	250 (57.6)	489 (56.27)		
N1	140 (32.18)	120 (27.65)	260 (29.92)		
N2	56 (12.87)	64 (14.75)	120 (13.81)		
**M stage**				3.012	0.083
M0	411 (94.48)	397 (91.47)	808 (92.98)		
M1	24 (5.52)	37 (8.53)	61 (7.02)		
TNM stage				5.858	0.119
I	58 (13.33)	73 (16.82)	131 (15.07)		
II	171 (39.31)	167 (38.48)	338 (38.90)		
III	182 (41.84)	158 (36.41)	340 (39.13)		
IV	24 (5.52)	36 (8.29)	60 (6.90)		
**Vascular tumor thrombus**				0.678	0.410
No	341 (78.39)	350 (80.65)	691 (79.52)		
Yes	94 (21.61)	84 (19.35)	178 (20.48)		
**Nerve involvement**				0.409	0.522
No	386 (88.74)	379 (87.33)	765 (88.03)		
Yes	49 (11.26)	55 (12.67)	104 (11.97)		
**MLH1**				0.024	0.877
Non-mutation	24 (5.52)	25 (5.76)	49 (5.64)		
Mutation	411 (94.48)	409 (94.24)	820 (94.36)		
**PMS2**				2.044	0.153
Non-mutation	37 (8.51)	26 (5.99)	63 (7.25)		
Mutation	398 (91.49)	408 (94.01)	806 (92.75)		
**MSH2**				0.659	0.417
Non-mutation	22 (5.06)	17 (3.92)	39 (4.49)		
Mutation	413 (94.94)	417 (96.08)	830 (95.51)		
**MSH6**				0.291	0.590
Non-mutation	15 (3.45)	18 (4.15)	33 (3.80)		
Mutation	420 (96.55)	416 (95.85)	836 (96.20)		
**MMR**				1.836	0.399
MSS	394 (90.57)	402 (92.63)	796 (91.60)		
MSI-L	11 (2.53)	6 (1.38)	17 (1.96)		
MSI-H	30 (6.90)	26 (5.99)	56 (6.44)		
**KRAS**				0.341	0.559
Non-mutation	238 (54.71)	246 (56.68)	484 (55.70)		
Mutation	197 (45.29)	188 (43.32)	385 (44.30)		
**BRAF**				0.125	0.724
Non-mutation	418 (96.09)	419 (96.54)	837 (96.32)		
Mutation	17 (3.91)	15 (3.46)	32 (3.68)		
**NRAS**				0.737	0.391
Non-mutation	422 (97.01)	425 (97.93)	847 (97.47)		
Mutation	13 (2.99)	9 (2.07)	22 (2.53)		
**PIK3CA**				0.625	0.429
Non-mutation	420 (96.55)	423 (97.47)	843 (97.01)		
Mutation	15 (3.45)	11 (2.53)	26 (2.99)		
**Leukocyte (10** ^**9**^ **/L)**	6.33 (5.23, 8.03)	6.3 (5.15, 8.09)	6.31 (5.19, 8.07)	−0.020	0.984
**Red blood cells (10** ^**12**^ **/L)**	4.27 (3.86, 4.64)	4.23 (3.86, 4.59)	4.25 (3.86, 4.61)	−0.844	0.399
**Platelets (10** ^**9**^ **/L)**	235 (196, 273)	232 (188, 286)	233 (193, 281)	−0.146	0.884
**Hemoglobin (g/L)**	126 (109, 139)	126 (109, 140)	126 (109, 139)	−0.052	0.958
**Neutrophil percentage**	0.62 (0.55, 0.71)	0.63 (0.54, 0.73)	0.62 (0.55, 0.72)	−0.030	0.976
**Lymphocytes percentage**	0.27 (0.19, 0.34)	0.27 (0.2, 0.34)	0.27 (0.19, 0.34)	−0.332	0.740
**Red blood cell distribution width (%)**	12.8 (12.2, 14)	12.8 (12.2, 13.93)	12.8 (12.2, 14)	−0.236	0.813

### Identification of risk factors for LNM in CRC patients

Comparative analysis comparing CRC patients with and without LNM revealed significant differences in various clinical factors (such as histological type, T-stage, M-stage, vascular tumor thrombus, nerve involvement, as well as genetic markers including PMS2, MSH2, KRAS, BRAF, PIK3CA, and counts of leukocytes and neutrophils) between the two groups (*P* < 0.05, as shown in Table 2). Conversely, gender, age, tumor site, tumor size, general classification, as well as markers such as MLH1, MSH6, MMR, NRAS, and hematological parameters including red blood cells, platelets, hemoglobin, lymphocytes, and red blood cell distribution width did not exhibit significant differences between the two patient groups (*P* > 0.05, as presented in Table 2). To further assess the relationships between the variables in the training set, Spearman’s rank correlation analysis was conducted. The results showed that most of the factors had correlation coefficients between −0.2 and 0.2, indicating a weak or negligible correlation between the majority of variables. A heatmap illustrating these correlations is presented in Fig. S1. The lack of strong correlations between variables suggests minimal multicollinearity, supporting the robustness of the model.

**Table 2:** Multivariate logistic regression analysis of risk factors associated with LNM in CRC patients within the training set.

**Clinical variables**	**CRC without LNM (*N* = 239)**	**CRC with LNM (*N* = 196)**	**Total (*N* = 435)**	**Statistics**	***P***
**Age (years)**	61.32 ± 12.59	59.96 ± 13.29	60.71 ± 12.91	−1.095	0.274
**Gender**				2.883	0.090
Male	137 (57.32)	128 (65.31)	265 (60.92)		
Female	102 (42.68)	68 (34.69)	170 (39.08)		
**Tumor size (cm)**	4.5 (3, 5.5)	4.5 (3.5, 5.5)	4.5 (3.5, 5.5)	−0.503	0.615
**Tumor site**				5.200	0.158
Rectum	96 (40.17)	98 (50.00)	194 (44.6)		
Left colon	71 (29.71)	55 (28.06)	126 (28.97)		
Right colon	58 (24.27)	34 (17.35)	92 (21.15)		
Other	14 (5.86)	9 (4.59)	23 (5.29)		
**General classification**				2.778	0.096
Ulcer type	154 (64.44)	141 (71.94)	295 (67.82)		
Raised type	85 (35.56)	55 (28.06)	140 (32.18)		
**Histological type**				26.665	<0.001
Poorly differentiated adenocarcinoma	6 (2.51)	6 (3.06)	12 (2.76)		
Moderately-poorly differentiated adenocarcinoma	25 (10.46)	48 (24.49)	73 (16.78)		
Moderately differentiated adenocarcinoma	192 (80.33)	129 (65.82)	321 (73.79)		
High-moderate differentiated adenocarcinoma	7 (2.93)	2 (1.02)	9 (2.07)		
High differentiated adenocarcinoma	5 (2.09)	0 (0)	5 (1.15)		
Other	4 (1.67)	11 (5.61)	15 (3.45)		
**T stage**				21.395	<0.001
T1	15 (6.28)	3 (1.53)	18 (4.14)		
T2	44 (18.41)	18 (9.18)	62 (14.25)		
T3	159 (66.53)	137 (69.9)	296 (68.05)		
T4	21 (8.79)	38 (19.39)	59 (13.56)		
**M stage**				4.791	0.029
M0	231 (96.65)	180 (91.84)	411 (94.48)		
M1	8 (3.35)	16 (8.16)	24 (5.52)		
**Vascular tumor thrombus**				28.113	<0.001
No	210 (87.87)	131 (66.84)	341 (78.39)		
Yes	29 (12.13)	65 (33.16)	94 (21.61)		
**Nerve involvement**				13.204	<0.001
No	224 (93.72)	162 (82.65)	386 (88.74)		
Yes	15 (6.28)	34 (17.35)	49 (11.26)		
**MLH1**				1.410	0.235
Non-mutation	16 (6.69)	8 (4.08)	24 (5.52)		
Mutation	223 (93.31)	188 (95.92)	411 (94.48)		
**PMS2**				8.972	0.003
Non-mutation	29 (12.13)	8 (4.08)	37 (8.51)		
Mutation	210 (87.87)	188 (95.92)	398 (91.49)		
**MSH2**				9.241	0.002
Non-mutation	19 (7.95)	3 (1.53)	22 (5.06)		
Mutation	220 (92.05)	193 (98.47)	413 (94.94)		
**MSH6**				0.863	0.353
Non-mutation	10 (4.18)	5 (2.55)	15 (3.45)		
Mutation	229 (95.82)	191 (97.45)	420 (96.55)		
**MMR**				4.583	0.101
MSS	210 (87.87)	184 (93.88)	394 (90.57)		
MSI-L	8 (3.35)	3 (1.53)	11 (2.53)		
MSI-H	21 (8.79)	9 (4.59)	30 (6.9)		
**KRAS**				4.732	0.030
Non-mutation	142 (59.41)	96 (48.98)	238 (54.71)		
Mutation	97 (40.59)	100 (51.02)	197 (45.29)		
**BRAF**				4.658	0.031
Non-mutation	234 (97.91)	184 (93.88)	418 (96.09)		
Mutation	5 (2.09)	12 (6.12)	17 (3.91)		
**NRAS**				2.615	0.106
Non-mutation	229 (95.82)	193 (98.47)	422 (97.01)		
Mutation	10 (4.18)	3 (1.53)	13 (2.99)		
**PIK3CA**				3.940	0.047
Non-mutation	227 (94.98)	193 (98.47)	420 (96.55)		
Mutation	12 (5.02)	3 (1.53)	15 (3.45)		
**Leukocyte (10** ^**9**^ **/L)**	6.76 (5.34, 9.24)	5.98 (5.13, 7.50)	6.33 (5.23, 8.03)	−3.724	<0.001
**Red blood cells (10** ^**12**^ **/L)**	4.23 ± 0.56	4.23 ± 0.63	4.23 ± 0.59	−0.068	0.946
**Platelets (10** ^**9**^ **/L)**	232 (190, 279)	237.5 (197.25, 270.75)	235 (196, 273)	−0.121	0.903
**Hemoglobin (g/L)**	125 (108, 138)	126.5 (109, 140)	126 (109, 139)	−0.813	0.416
**Neutrophil percentage**	0.6 (0.56, 0.67)	0.66 (0.54, 0.77)	0.62 (0.55, 0.71)	−2.600	0.009
**Lymphocytes percentage**	0.27 (0.18, 0.34)	0.27 (0.20, 0.34)	0.27 (0.19, 0.34)	−1.161	0.872
**Red blood cell distribution width (%)**	12.8 (12.3, 14.1)	12.8 (12.2, 13.8)	12.8 (12.2, 14)	−0.323	0.747

**Figure 1:**
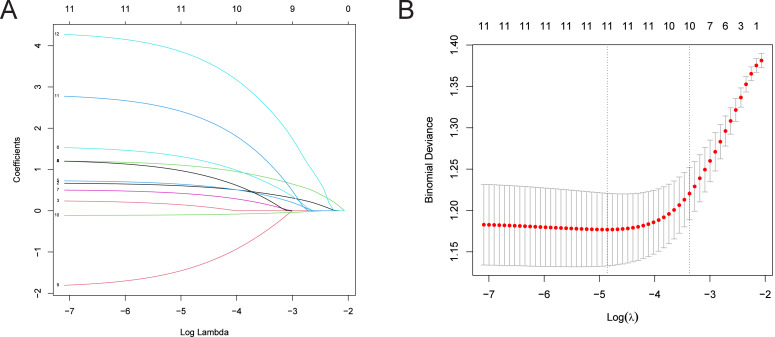
LASSO regression analysis for identifying risk factors in CRC patients with LNM. (A) Distribution of coefficients for independent variables, with the number of predictors displayed based on their non-zero coefficients at various *λ* values. (B) Cross-validation error plot. Note: While the model includes 12 risk factors, one variable was reduced to zero during the LASSO process at the selected *λ*, resulting in 11 visible lines in panel A. This is a standard outcome of LASSO regression where some predictors may not contribute at specific levels of regularization.

LASSO regression analysis was utilized to further refine the factors influencing LNM of CRC patients (Simon et al., 2011; Tibshirani, 1997). The “glnmnet” R package was used for variable selection and shrinkage (Friedman, Hastie & Tibshirani, 2010). The independent variables in LASSO regression are a matrix of clinically relevant candidate factors, while the response variable represents the occurrence of LNM in the training set. The penalty parameter (*λ*) of the model was determined by ten-fold cross-validation. The findings indicate that with 11 variables, the binomial deviance value of the regression model is minimized. The selected variables at this point include: T stage, M stage, vascular tumor thrombus, nerve involvement, PMS2, MSH2, KRAS, BRAF, PIK3CA, leukocyte count, and neutrophil count (Fig. 1).

### Development of nomogram for predicting LNM in CRC patients

Multivariate analysis was conducted on the 11 single-factor variables identified through LASSO regression. A binary logistic regression model was then established to predict the incidence of LNM, using all 11 selected variables. The model is expressed as:

\begin{eqnarray*}\text{logit} \left( p \right) =-5.645+0.638\cdot \mathrm{T}~\text{stage}+0.213\cdot \mathrm{M}~\text{stage}+1.194\cdot \text{Vascular tumor}\nonumber\\\displaystyle \mathrm{thrombus}+0.701\cdot \text{Nerve involvement}+1.250\cdot \mathrm{PMS}2+1.572\cdot \mathrm{MSH}2+0.533\cdot \text{KRAS}+\nonumber\\\displaystyle 1.687\cdot \text{BRAF}-1.649\cdot \mathrm{PIK}3\mathrm{CA}-0.145\cdot \text{Leukocyte}+2.093\cdot \text{Neutrophils} \end{eqnarray*}

The results revealed that T stage, vascular tumor thrombus, PMS2, MSH2, KRAS, BRAF, PIK3CA, leukocyte, and neutrophil were independent predictors for the incidence of LNM (all with *P* < 0.05, as shown in Table 3). The Hosmer-Lemeshow test chi-square value was 10.206, and the significance was 0.251, indicating that the model had good goodness of fit. For each 1-unit increase in T-stage, the likelihood of lymph node metastasis (LNM) is associated with a 1.892-fold increase. The presence of vascular tumor thrombus is associated with a 3.301-fold higher risk of LNM compared to its absence. Mutations in PMS2, MSH2, KRAS, and BRAF are associated with a 3.489, 4.815, 1.704, and 5.402-fold increased risk of LNM, respectively, compared to non-mutation. Each additional unit of neutrophil count is associated with an 8.113-fold increase in the likelihood of LNM. The presence of a PIK3CA mutation is associated with a 0.192-fold reduced risk of LNM compared to non-mutation. Lastly, for each 1-unit increase in leukocyte count, the likelihood of LNM is associated with a 0.865-fold decrease.

**Table 3:** Comparative analysis of risk factors for LNM among CRC patients in the training set.

Independent variable	β	SE	Wals	*P*	OR (95% CI)
T stage	0.638	0.183	12.126	<0.001	1.892 (1.322–2.709)
M stage	0.213	0.513	0.172	0.678	1.237 (0.453–3.38)
Vascular tumor thrombus	1.194	0.285	17.548	<0.001	3.301 (1.888–5.773)
Nerve involvement	0.701	0.377	3.463	0.063	2.016 (0.963–4.219)
PMS2	1.25	0.458	7.433	0.006	3.489 (1.421–8.567)
MSH2	1.572	0.653	5.788	0.016	4.815 (1.338–17.329)
KRAS	0.533	0.222	5.771	0.016	1.704 (1.103–2.632)
BRAF	1.687	0.705	5.725	0.017	5.402 (1.357–21.509)
PIK3CA	−1.649	0.768	4.619	0.032	0.192 (0.043–0.865)
Leukocyte	−0.145	0.041	12.463	<0.001	0.865 (0.798–0.938)
Neutrophils	2.093	0.913	5.256	0.022	8.113 (1.355–48.578)
Intercept	−5.645	1.149	24.151	<0.001	0.004

**Figure 2:**
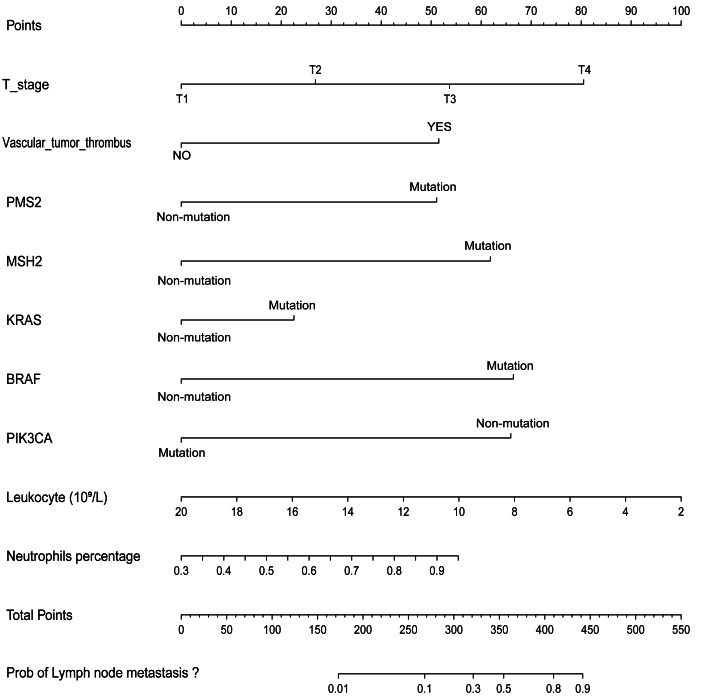
Nomogram for predicting the probability of LNM in CRC patients.

**Figure 3:**
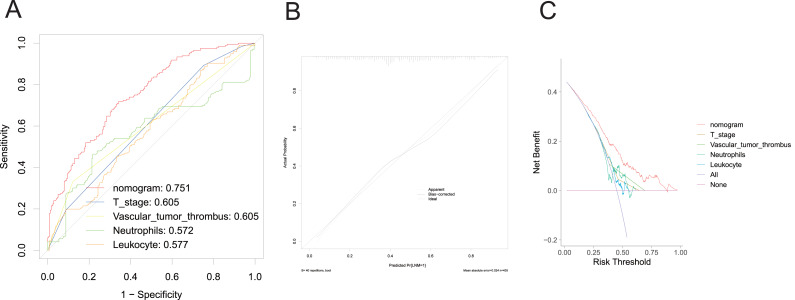
Evaluation of the performance of nomogram in the training set. ROC curve (A), calibration curve (B), and DCA curve (C).

A nomogram was developed to clinically predict LNM using independent predictors (T stage, vascular tumor thrombus, PMS2, MSH2, KRAS, BRAF, PIK3CA, leukocyte count, and neutrophil count) identified from a multivariate logistic regression model (Fig. 2). Among these predictors, T stage (AUC: 0.605), vascular tumor thrombus (AUC: 0.605), leukocyte (AUC: 0.577), and neutrophil (AUC: 0.572) exhibited the highest AUC values. Notably, the predictive performance of the nomogram (AUC: 0.751) surpassed that of individual variables (Fig. 3A). The concordance index (C-index) was calculated as 0.751 (95% CI [0.728–0.774]), indicating good predictive ability. The calibration curve (Fig. 3B) closely matches the reference line, suggesting excellent concordance between predicted and observed values of the nomogram. Decision curve analysis (DCA) revealed that the nomogram provided greater net clinical benefit compared to a single clinicopathological feature, as evidenced by its deviation from the reference lines (Fig. 3C), indicating its superiority in clinical prediction for patients.

### Validation of nomogram for predicting LNM in CRC patients

**Figure 4:**
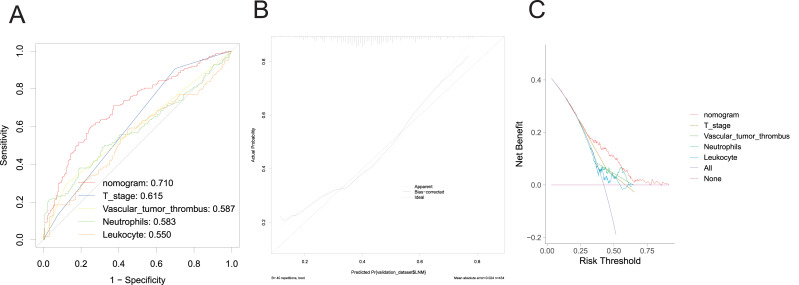
Evaluation of the performance of nomogram in the validation set. ROC curve (A), calibration curve (B), and DCA curve (C).

In the validation group, the nomogram exhibited robust predictive performance for LNM. The validation set ROC curve (Fig. 4A) showcased the model’s discriminative prowess in distinguishing patients with and without LNM. Notably, the nomogram achieved an AUC value of 0.710, surpassing individual clinical indicators, underscoring its good discriminatory ability. The calibration curve (Fig. 4B) further underscored the alignment between predicted probabilities and observed outcomes, indicating the model’s reliability. Additionally, the DCA curve (Fig. 4C) depicted the clinical utility of the model across various threshold probabilities, demonstrating enhanced net benefit compared to single clinicopathological factors. These findings collectively underscore the robust discriminative power, calibration, and significant clinical utility of the prediction model. They suggest its potential to improve accurate risk assessment and guide treatment decisions for CRC patients with LNM, thereby profoundly impacting clinical decision-making.

## Discussion

CRC remains an important public health problem worldwide, and LNM is an important prognostic factor for CRC. LNM in CRC is a multifaceted process involving tumor cell invasion, endocytosis, transport through the lymphatic system, retention in the lymph nodes, extravasation, proliferation, angiogenesis, and interaction with the immune system (Cao et al., 2022; Huang & Chen, 2017; Jiang et al., 2013; Wang et al., 2021; Yan, Su & Qin, 2020). Understanding the risk factors associated with LNM is essential to guide clinical management and improve patient prognosis. Analysis of CRC risk factors revealed some noteworthy findings T stage, vascular tumor thrombus, PMS2, MSH2, KRAS, BRAF, PIK3CA, leukocyte, and neutrophils emerged as important risk factors for LNM in CRC. In contrast, leukocytes showed a protective effect against metastasis.

This study revealed a direct correlation between the probability of LNM in CRC patients and the advancement of T-stage. Multivariate analysis demonstrated that the risk of LNM increased by a factor of 1.892 with each unit increase in T-stage, consistent with findings from prior research (Hashmi et al., 2018). Additionally, vascular tumor thrombus emerged as an independent predictor of LNM. Patients with CRC exhibiting vascular tumor thrombus were found to be 3.3 times more likely to develop metastasis compared to those without such thrombus presence. Vascular tumor thrombus, also known as vascular infiltration, is the presence of tumor cells in the lumen of a blood vessel or the disruption of the vessel wall by tumor cells, which is the first stage of metastasis formation (Li et al., 2023). In gastric cancer, lymphovascular invasion is a significant independent risk factor for LNM (Hu et al., 2019). The rate of LNM was higher in CRC with vascular tumor thrombosis than in those without LNM (Li et al., 2020). The ascending colon and rectum are the most common tumor sites for tumor thrombosis. Many studies have concluded that vascular tumor thrombosis is a very important risk factor for LNM, and the presence of tumor thrombosis in the primary tumor indicates the possibility of tumor cells metastasizing with blood vessels (Chung et al., 2016; Fu et al., 2021; Yu et al., 2009).

PMS2 is involved in encoding the pair-mismatch repair system, and mutations in PMS2 are associated with hereditary nonpolyposis colorectal cancer (HNPCC) (Yurgelun et al., 2017). In a clinical study, KRAS/NRAS mutations were identified in 316 (42.6%) CRC patients, and BRAF mutations were identified in 47 (7.2%) CRC patients (Yurgelun et al., 2017). It has been suggested that deletion of MSH-2 and MSH-6 expression is associated with right colon location, dysplasia, and mucinous differentiation (Karahan et al., 2015), and our results pointed out that MSH-2 positivity was associated with a 4.815-fold increase in the risk of LNM. However, this is contrary to the results of our study. However, this finding contrasts with the typical association between MSH-2 expression and microsatellite instability-high (MSI-H) status, which is often linked to a better prognosis and lower incidence of LNM in CRC (Kang et al., 2018). MSI-H CRCs, characterized by a deficiency in the mismatch repair (MMR) system, are known to have higher mutational burdens, which can influence tumor progression and metastatic potential (Taieb et al., 2022). While studies on MSH-2 in CRC LNM are limited, the relationship between MMR status and LNM risk remains an important area for further research. Specific screening for these mutations minimizes the risk of syndrome-specific cancers (Giardiello et al., 2014; Shia, 2008; Syngal et al., 2015). Furthermore, the presence of high levels of neutrophils in the tumor microenvironment is associated with tumor progression and metastasis (Gonzalez, Hagerling & Werb, 2018; Ocana et al., 2017). In contrast, leukocytes play a role in immune surveillance and may play a protective role against metastatic spread (Mitchell & King, 2015; Mueller, 2022; Olson & Ley, 2002). Higher leukocyte levels may indicate a stronger immune response against tumor cells, thus limiting their ability to metastasize to regional lymph nodes. Understanding these risk factors provides valuable insights for risk stratification, treatment selection, and the development of targeted therapies aimed at preventing or attenuating LNM in CRC patients.

The utilization of LASSO regression for variable selection and model construction in this study conferred several advantages, notably its capability to manage high-dimensional data and alleviate issues related to multicollinearity. By penalizing the absolute magnitude of the regression coefficients, LASSO regression encourages model sparsity, resulting in simpler and more interpretable models. In addition, the regularization property of LASSO helps prevent overfitting, making the model more robust and generalizable to new data.

However, there are still limitations of the study that are worth considering. The scarcity of PIK3CA-positive samples raises concerns about the representativeness of the study results, especially regarding the role of PIK3CA mutations in CRC metastasis. In addition, while LASSO regression is effective for variable selection, it may not capture the complex nonlinear relationships between predictor variables and outcomes. Alternative modeling techniques or combinations of interactions between variables could enhance the predictive performance of the model.

In conclusion, the findings of this study significantly enhance our comprehension of the intricate interactions among diverse factors in the context of LNM in CRC. By utilizing LASSO regression and developing a column-line graphical model, this study provides clinicians with a valuable tool for personalized risk assessment and treatment decision-making for CRC patients. However, further studies are needed to validate and refine the model for broader clinical applications.

## Conclusion

The study identified pivotal risk factors associated with LNM in CRC, including T stage, presence of vascular tumor thrombus, genetic markers (PMS2, MSH2, KRAS, BRAF), and neutrophil counts, while leukocytes displayed a protective effect. These findings underscore the importance of comprehensive risk assessment and personalized treatment strategies in CRC management. Integrating these factors into clinical decision-making empowers clinicians to optimize patient care, thereby enhancing outcomes and tailoring interventions for individuals with CRC.

##  Supplemental Information

10.7717/peerj.19148/supp-1 Supplemental Information 1 Heat map of the correlation coefficients

10.7717/peerj.19148/supp-2 Supplemental Information 2 Convert factors

10.7717/peerj.19148/supp-3 Supplemental Information 3 Code

10.7717/peerj.19148/supp-4 Supplemental Information 4 Raw data

## Additional Information and Declarations

### Data Availability

The following information was supplied regarding data availability: The raw data are available in the Supplemental File.
