# Supplementary figures and images for: LASSO-derived nomogram prediction model for lymph node metastasis in colorectal cancer: a retrospective analysis
Source: PeerJ. 2025 Apr 14;13:e19148. doi: 10.7717/peerj.19148 (PMC12005177; doi:10.7717/peerj.19148)

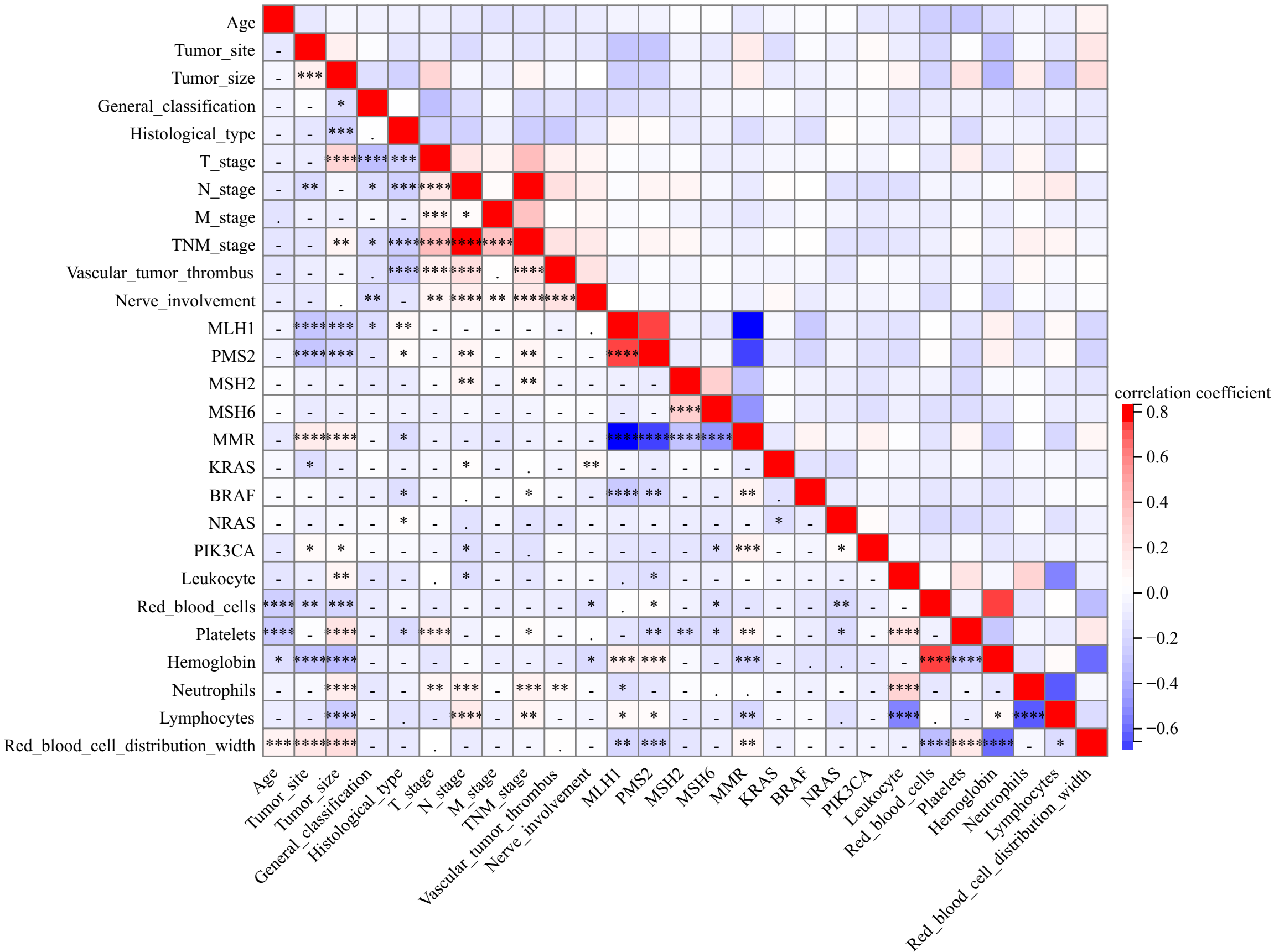

Supplement: Supplemental Information 1 [file peerj-13-19148-s001.pdf]
